# The Mitochondrial Voltage-Dependent Anion Channel 1, Ca^2+^ Transport, Apoptosis, and Their Regulation

**DOI:** 10.3389/fonc.2017.00060

**Published:** 2017-04-10

**Authors:** Varda Shoshan-Barmatz, Soumasree De, Alon Meir

**Affiliations:** ^1^Department of Life Sciences, National Institute for Biotechnology in the Negev, Ben-Gurion University of the Negev, Beer-Sheva, Israel

**Keywords:** apoptosis, Ca^2+^ transporters, mitochondria, oligomerization, voltage-dependent anion channel

## Abstract

In the outer mitochondrial membrane, the voltage-dependent anion channel 1 (VDAC1) functions in cellular Ca^2+^ homeostasis by mediating the transport of Ca^2+^ in and out of mitochondria. VDAC1 is highly Ca^2+^-permeable and modulates Ca^2+^ access to the mitochondrial intermembrane space. Intramitochondrial Ca^2+^ controls energy metabolism by enhancing the rate of NADH production *via* modulating critical enzymes in the tricarboxylic acid cycle and fatty acid oxidation. Mitochondrial [Ca^2+^] is regarded as an important determinant of cell sensitivity to apoptotic stimuli and was proposed to act as a “priming signal,” sensitizing the organelle and promoting the release of pro-apoptotic proteins. However, the precise mechanism by which intracellular Ca^2+^ ([Ca^2+^]_i_) mediates apoptosis is not known. Here, we review the roles of VDAC1 in mitochondrial Ca^2+^ homeostasis and in apoptosis. Accumulated evidence shows that apoptosis-inducing agents act by increasing [Ca^2+^]_i_ and that this, in turn, augments VDAC1 expression levels. Thus, a new concept of how increased [Ca^2+^]_i_ activates apoptosis is postulated. Specifically, increased [Ca^2+^]_i_ enhances VDAC1 expression levels, followed by VDAC1 oligomerization, cytochrome *c* release, and subsequently apoptosis. Evidence supporting this new model suggesting that upregulation of VDAC1 expression constitutes a major mechanism by which apoptotic stimuli induce apoptosis with VDAC1 oligomerization being a molecular focal point in apoptosis regulation is presented. A new proposed mechanism of pro-apoptotic drug action, namely Ca^2+^-dependent enhancement of VDAC1 expression, provides a platform for developing a new class of anticancer drugs modulating VDAC1 levels *via* the promoter and for overcoming the resistance of cancer cells to chemotherapy.

## Overview

Intracellular Ca^2+^concentration ([Ca^2+^]_i_) regulates a number of cellular and intercellular events, such as the cell cycle, proliferation, gene transcription, and cell death pathways, as well as processes like muscle contractility and neuronal processing and transmission (1). The alteration of Ca^2+^ homeostasis is closely related with various cancer hallmarks, including proliferation, migration, angiogenesis, invasion abilities, and resistance to cell death (2).

Various systems and mechanisms have evolved to control and respond to minute changes in Ca^2+^ concentrations and localization (3). Moreover, many cellular compartments participate in the Ca^2+^ signaling network regulation. [Ca^2+^]_i_ is controlled *via* its transport in and out of the cell or/and in and out of intracellular organelles. Within a given compartment, [Ca^2+^] can be buffered by binding to specific proteins and other molecules, as well as existing in its free form, albeit differentially across compartments (1). The major organelles that participate in controlling Ca^2+^ dynamics include the endoplasmic reticulum (ER) and mitochondria (4). Imbalance in the control of [Ca^2+^]_i_ can lead to mitochondria Ca^2+^ overload and ultimately, to toxic effects. Tumor cells exhibit a well-developed capacity for modulating cytosolic Ca^2+^ levels by remodeling the cellular machinery that participates in processes that determine Ca^2+^ dynamics and homeostasis, as well as changes in sensitivity to the induction of cell death. This review is focused on the mitochondrial gatekeeper protein voltage-dependent anion channel 1 (VDAC1) and its role in Ca^2+^ transport and on Ca^2+^-mediated apoptosis involving regulation of VDAC1 expression levels.

## Ca^2+^ and Mitochondria

Mitochondria not only play a key role in metabolism but also serve as a major hub for cellular Ca^2+^ homeostasis, regulating oxidative phosphorylation (OXPHOS) (4–7) and modulating cytosolic Ca^2+^ signals (8, 9), cell death (10), and secretion (11, 12). Enclosed by two different membranes, namely the outer mitochondrial membrane (OMM) and the inner mitochondrial membrane (IMM), mitochondria thus present two aqueous compartments, the intermembrane space (IMS) and the matrix (M). To reach the matrix, Ca^2+^ must cross both the OMM and the IMM. Indeed, the mitochondrial matrix is one of the major cellular Ca^2+^ stores or buffers and is used to control [Ca^2+^]_i_ and dynamics. Within the mitochondrial matrix, Ca^2+^ is precipitated as insoluble CaPO_4_, which exists in equilibrium with free Ca^2+^ (7, 13).

It is well established that mitochondria can rapidly sequester large and sudden increases in [Ca^2+^]_i_ at the expense of the membrane potential across the IMM that is generated by the electron transport chain (6). Intramitochondrial Ca^2+^ controls energy metabolism by enhancing the rate of NADH production *via* modulating critical enzymes, such as those of the tricarboxylic acid (TCA) cycle and fatty acid oxidation (14, 15), linking glycolysis to the TCA cycle (16). Indeed, matrix Ca^2+^ is an essential cofactor for several rate-limiting TCA enzymes, namely pyruvate dehydrogenase, isocitrate dehydrogenase, and α-ketoglutarate dehydrogenase.

Mitochondrial Ca^2+^ ([Ca^2+^]_m_) homeostasis is important not only for energy production but also for regulating [Ca^2+^]_i_ and activating cell apoptotic pathways (10, 17). Several recent reviews have discussed the basic principles that govern [Ca^2+^]_m_ homeostasis and maintenance of Ca^2+^ dynamics within organelles (18–22). The OMM and IMM pathways allowing Ca^2+^ entry into and exit from mitochondria are presented below.

## Pathways Mediating Ca^2+^ Fluxes in the Mitochondria

Several mitochondria membrane proteins play central roles in Ca^2+^ signaling and/or Ca^2+^ influx and efflux in normal and disease conditions. Ca^2+^ transport across the IMM is mediated *via* several proteins, including the mitochondrial Ca^2+^ uniporter (MCU) (23, 24) and the Na^+^/Ca^2+^ exchanger, NCLX, the major Ca^2+^ efflux mediator (25, 26). In the OMM, VDAC1 was shown to control Ca^2+^ permeability (27–30).

### VDAC1, the Ca^2+^ Channel in the OMM

Three different isoforms of VDAC have been identified, VDAC1, VDAC2, and VDAC3. VDAC1 has been best studied, whereas only limited information regarding the cellular functions of VDAC2 and VDAC3 is available (31). Thus, we focus here on VDAC1.

#### VDAC1, a Multifunctional Channel, Controls Cell Metabolism

**Figure 1:**
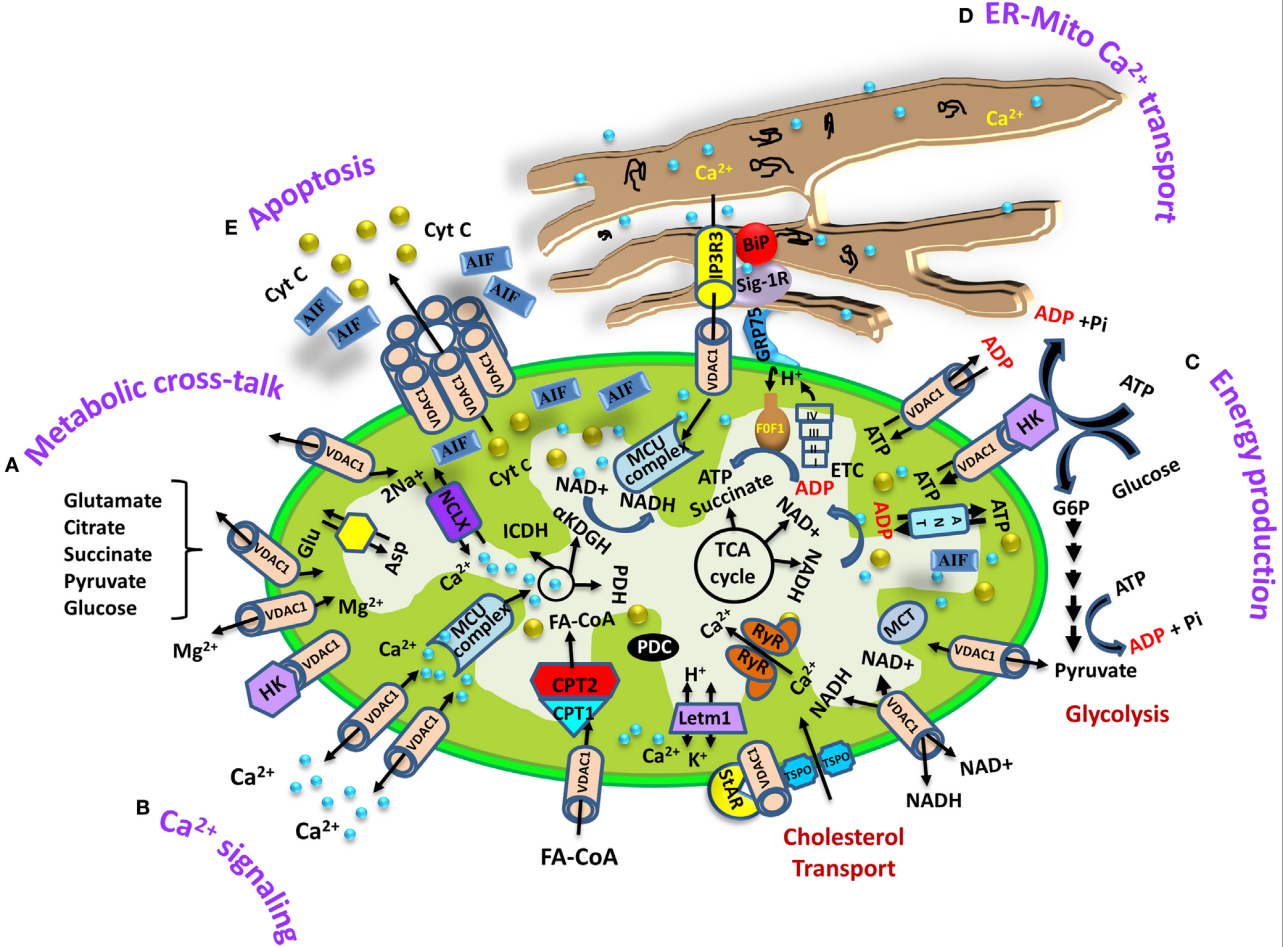
**Schematic representation of voltage-dependent anion channel 1 (VDAC1) as a multifunctional protein involved in Ca^2+^ and metabolite transport, energy production, and the structural and functional association of mitochondria with the endoplasmic reticulum (ER)**. The various functions of VDAC1 in cell and mitochondria functions are presented. These include **(A)** Ca^2+^ signaling by transporting Ca^2+^; **(B)** control of metabolic cross talk between the mitochondria and the rest of the cell; **(C)** mediating cellular energy production by transporting ATP/ADP, NADH, and acyl-CoA from the cytosol to the intermembrane space and regulating glycolysis *via* the association with hexokinase (HK); **(D)** involvement in structural and functional association with the ER, mediating Ca^2+^ transport from the ER to mitochondria; **(E)** participation in apoptosis *via* its oligomerization to form a protein-conducting channel within a VDAC1 homo-oligomer, allowing Cyto *c* release and apoptotic cell death. Ca^2+^ influx and efflux transport systems in the outer mitochondrial membrane (OMM) and IMM are shown. In the OMM, VDAC1 is presented as a Ca^2+^ channel and also functions in the transport of Mg^2+^. In the IMM, Ca^2+^ uptake into the matrix is mediated by a Ca^2+^-selective transporter, the mitochondrial Ca^2+^ uniporter (MCU), regulated by a calcium-sensing accessory subunit (MCU1). Ryanodine receptor (RyR) in the IMM mediates Ca^2+^ influx. Ca^2+^ efflux is mediated by NCLX, an Na^+^/Ca^2+^ exchanger. High levels of matrix Ca^2+^ accumulation trigger the opening of the PTP, a fast Ca^2+^ release channel. The function of Ca^2+^ in regulation of energy production is mediated *via* tricarboxylic acid (TCA) cycle regulation. This includes activation of pyruvate dehydrogenase (PDH), isocitrate dehydrogenase (ICDH), and α-ketoglutarate dehydrogenase (αKGDH) by intramitochondrial Ca^2+^, leading to enhanced activity of the TCA cycle. The electron transport chain (ETC) and the ATP synthase (F_o_F_1_) are also presented. Molecular fluxes are indicated by arrows. VDAC1 mediates the transfer of fatty acid acyl-CoAs across the OMM to the IMS, where they are converted into acylcarnitine by CPT1a for further processing by β-oxidation. VDAC1 is involved in cholesterol transport by being constituent of a multi-protein complex, the transduceosome, containing Star/TSPO/VDAC1. The ER associated with the mitochondria is presented with key proteins indicated. These include the inositol 3 phosphate receptor type 3 (IP3R3), the sigma1 receptor (Sig1R) (a reticular chaperone), binding immunoglobulin protein (BiP), the ER heat shock protein (HSP70) chaperone, and glucose-regulated protein 75 (GRP75). IP3 activates the IP3R in the ER to release Ca^2+^ that is directly transferred to the mitochondrion *via* VDAC1.

VDAC1 at the OMM controls metabolic cross talk between mitochondria and rest of the cell by allowing the entry of metabolites (pyruvate, malate, succinate, nucleotides, and NADH) into the mitochondria and the exit of newly formed molecules, such as hemes (32, 33) (Figure 1). VDAC1 is also involved in cholesterol transport and mediates the fluxes of ions, including Ca^2+^ (34), serves as a ROS transporter, and contributes to regulating the redox states of mitochondria and the cytosol (32–34). Moreover, VDAC1 at the OMM interacts with proteins that mediate and regulate the integration of mitochondrial functions with other cellular activities. VDAC1 forms a complex with adenine nucleotide translocase (ANT) and creatine kinase (35). The interaction of VDAC1 with hexokinase (HK) allows for coupling between OXPHOS and glycolysis, an important factor in cancer cell energy homeostasis (the Warburg effect) (36). Thus, VDAC1 appears to be a convergence point for a variety of cell survival and death signals, mediated through its association with various ligands and proteins.

#### VDAC1 As Ca^2+^ Transporter at OMM

Found in the OMM, VDAC1 regulates the transport of Ca^2+^ in and out of the mitochondria. VDAC1 is highly Ca^2+^-permeable and modulates the accessibility of Ca^2+^ to the IMS (27–30) (Figure 1). Bilayer-reconstituted VDAC1 under voltage-clamp conditions and in the presence of different CaCl_2_ concentration gradients showed well-defined voltage-dependent channel conductance as observed with either NaCl or KCl solution (27, 29). Bilayer-reconstituted VDAC1 showed higher permeability to Ca^2+^ in the low conductance state (29). The Ca^2+^ permeability of VDAC1 has also been established upon VDAC1 reconstitution into liposomes (27).

Various studies support the function of VDAC1 in the transport of Ca^2+^ and in cellular Ca^2+^ homeostasis. VDAC1 overexpression increases [Ca^2+^]_m_ concentration in HeLa cells and skeletal myotubes (37), and silencing of VDAC1 expression by siRNA attenuates [Ca^2+^]_m_ uptake and cell apoptosis induced by H_2_O_2_ or ceramide (38). It was also proposed that the magnitude of Ca^2+^ transfer into the mitochondrial matrix is regulated by protein–protein interactions between Bcl-xL and VDAC1 or VDAC3, with this interaction promoting matrix Ca^2+^ accumulation by increasing Ca^2+^ transfer across the OMM (39).

Silencing each of the VDAC isoforms in the presence of a pro-apoptotic stimulus revealed that each was differentially sensitive to H_2_O_2_, with VDAC1 silencing potentiating H_2_O_2_-induced apoptosis and impairing [Ca^2+^]_m_ loading, while VDAC2 silencing had the opposite effects (38). In addition, several VDAC-interacting molecules like 4,4′-diisothiocyanostilbene-2,2′-disulfonic acid (DIDS), 4-acetamido-4′-isothiocyanato-stilbene-2,2′-disulfonic acid, and dinitrostilbene-2,2′-disulfonic acid were shown to prevent apoptosis and also inhibit the rise in [Ca^2+^] levels associated with apoptosis induction (40). In another example, 5-aminolevulinic precluded Ca^2+^-mediated oxidative stress and apoptosis through VDAC1 inhibition (41).

#### VDAC1 Possesses Ca^2+^-Binding Sites

Several lines of evidence suggest that VDAC1 possesses divalent cation-binding site(s). [Ca^2+^], at micromolar concentrations, switched VDAC1 from a low to high conductance state (30). The trivalent ions La^3+^ and Tb^3+^, known to bind to Ca^2+^-binding proteins (42), reduced the channel conductance of bilayer-reconstituted VDAC (43). This and the direct demonstration of Tb^3+^ binding to purified VDAC1, as reflected in an enhanced green fluorescence, further suggest that VDAC1 possesses divalent cation-binding site(s) that its occupation by La^3+^ or Tb^3+^ lead to reduced channel conductance.

Similarly, molecules known to specifically interact with several Ca^2+^-binding proteins like ruthenium red (RuR) (43) and ruthenium amine binuclear complex (Ru360) (44), as well as a photo-reactive analog, azido ruthenium (AzRu) (45), induced VDAC1 channel closure in a time-dependent manner and stabilized the channel in a low conducting state. These compounds also inhibited apoptosis (43–45).

The putative VDAC1 metal binding site for RuR and AzRu was analyzed by mutation of specific VDAC1 residues (43). It has been demonstrated that E72 and E202 are essential for RuR-mediated reduction of bilayer-reconstituted VDAC1 conductance and for RuR-mediated protection against VDAC1-induced cell death (43, 46). This suggests that these two glutamate residues, located in two different β-strands, may form the VDAC1 Ca^2+^-binding site(s), or part thereof. However, their distant location and their being located in transmembrane sequences, suggest that these residues may stabilize VDAC1 in a conformation that is recognized by RuR and AzRu. Thus, these Ru-containing molecules may bind to a non-defined site in VDAC1 to induce conformation changes leading to reduced conductance and inhibited apoptosis.

The competition between Ca^2+^ and RuR (43), as well as the demonstration of VDAC gating regulation by physiological levels of Ca^2+^, whereby Ca^2+^ increases the conductance of the VDAC1 channel (30), supports the physiological function of the VDAC1 Ca^2+^-binding site(s).

#### VDAC1 Protein–Protein Interactions Regulate [Ca^2+^]_m_ Transport

Interactions between VDAC1 and Bcl-2 family proteins, such as Bax/Bak, Bcl-2, and Bcl-xL, mediating the regulation of apoptosis, are well documented (33, 47–55). It has been shown that interaction of Bcl-xL with VDAC1 or VDAC3 promoted [Ca^2+^]_m_ uptake (39). It was also reported that all three VDAC isoforms interact with regulator of microtubule dynamics protein 3 (56), a protein at the OMM involved in [Ca^2+^]_i_ homeostasis regulation (57, 58). VDAC1 interacts with endothelial NO synthase (eNOS), with such interaction amplifying eNOS activity in a [Ca^2+^]_i_-mediated manner (59). VDAC also interacts with the L-type Ca^2+^ channel, and it was suggested that impaired communication between the L-type Ca^2+^ channel and mitochondrial VDAC contributes to cardiomyopathy (60). Thus, such interactions of VDAC with proteins associated with Ca^2+^ transport or activated by Ca^2+^ point to VDAC as functioning not only in [Ca^2+^]_i_ homeostasis but also in many Ca^2+^-regulated cellular activities.

#### VDAC1 Function in Mitochondria—ER/Sarcoplasmic Reticulum (SR) Ca^2+^-Cross Talk

The participation of VDAC1 in supramolecular complexes and intracellular communication, including Ca^2+^ signal delivery between the ER and mitochondria, was postulated over a decade ago (28, 61, 62). The components involved in ER–mitochondria interaction include the IP_3_ receptor and grp75 on the ER as tethering components and VDAC1 on the OMM (63). VDAC1 (but not VDAC2 or VDAC3) was found to provide the route for Ca^2+^ entry into mitochondria upon apoptotic stimulus, representing a fundamental factor in mitochondria physiology (38). It was, moreover, proposed that the magnitude of Ca^2+^ transfer from the ER into the mitochondrial matrix is regulated by Bcl-xL (39, 51). ER–mitochondria cross talk regulates not only Ca^2+^ transfer but also different processes, such as mitochondrial fission, autophagy, and inflammation (64). Finally, Ca^2+^ dynamics are greatly enhanced where there is close apposition of the ER with mitochondria, as compared to the bulk cytosol. Such changes in Ca^2+^ signal profiles were modified by ROS, as monitored with genetically encoded redox indicators (65).

### MCU and Auxiliary Subunits Form a Selective Ca^2+^ Transporter in the IMM

Ca^2+^ transport across the IMM and into the matrix is mediated *via* the MCU (23, 24), with the driving force being the steep mitochondrial membrane potential (8, 66, 67) (Figure 1). Such delivery is inhibited by RuR and its derivative, Ru360 (68).

The major channel-forming subunit of the MCU complex (CCDC109A) consists of two transmembrane and the N-terminal domains and forms a complex in the IMM with many gatekeeper membrane proteins (23, 24, 69–71). The calcium-sensing accessory subunits MICU1, MICU2, and MCUb are proposed to serve as negative regulators, while mitochondrial Ca^2+^ uniporter regulator 1 (MCUR1), essential MCU regulator, and SLC25A23 are essential for MCU activity (72–76). MCUR1 may, however, also play other roles, such as in cytochrome *c* oxidase assembly (77), as a cytosolic Ca^2+^-buffering agent (78), or in ROS generation (79).

The functional role of MCU under physiological conditions was extensively studied using several silencing techniques (80–85). Interestingly, MCU knockout mice did not exhibit obvious defects in mitochondrial number or morphology and any physiological function (82, 86, 87). MCU deletion was found to be lethal for C57BL/6 mice, whereas knockout mice with an outbred CD1 background were viable, albeit with reduced numbers (88). Basal organ functions were maintained, and impairment was only observed in the physiological adaptation of skeletal muscle to exercise (82). In cardiac-specific conditional MCU-deficient mice, the heart displayed increased resistance to ischemia–reperfusion injury (87, 89).

### Na^+^/Ca^2+^ Exchanger Function in Ca^2+^ Efflux and Its Regulation

Mitochondrial Ca^2+^ is mainly determined by the balance between influx through the MCU and efflux *via* NCLX (90). To restore resting [Ca^2+^]_m_ levels, Ca^2+^ efflux across IMM is mediated by the Na/Ca/Li exchanger (NCLX) or Na^+^/Ca^2+^ exchanger (25, 26, 91), and possibly by Letm1, under certain conditions, which functions as a Ca^2+^/H^+^ anti-porter in addition to being a H^+^/K^+^ anti-porter (see Other Proteins Proposed as Participating in or Mediating Ca^2+^ Efflux from Mitochondria) (92). NCLX mediates efflux of Ca^2+^ from the mitochondrial matrix to the IMS (20, 25, 93–95). In contrast to the plasma membrane Na^+^/Ca^2+^ exchanger, NCLX transports Li^+^ ions in addition to Na^+^ and Ca^2+^ (96). NCLX has also been proposed to regulate Ca^2+^-induced NAD(P)H production and matrix redox state modulation (97).

Mitochondrial Ca^2+^ regulates heart metabolism, where steady-state [Ca^2+^]_m_ is determined by the dynamic balance between MCU-based Ca^2+^ influx and NCLX-based Ca^2+^ efflux (98). It has been proposed that a novel role of NCLX is to regulate the automaticity of cardiomyocytes *via* modulating SR Ca^2+^ handling (99). NCLX has been proposed to be involved in several pathological conditions. In ischemia, NCLX acts as a key regulator of [Ca^2+^]_m_ accumulation (100), while in diabetic cardiac myocytes, NCLX is more susceptive to changes in the outside (cytosolic) Na^+^ concentration, as compared with controls (101). Phosphorylation of NCLX has been reported to reverse Ca^2+^ mitochondrial overload and promote survival of PINK1-deficient dopaminergic neurons (102).

### Other Proteins Proposed as Participating in or Mediating Ca^2+^ Efflux from Mitochondria

The transient opening of the mitochondrial permeability transition pore (MPTP or PTP) represents another mechanism for Ca^2+^ release from mitochondria. However, its function is probably related to non-physiological Ca^2+^ overload that depolarizes mitochondria by an irreversible opening of the PTP, leading to apoptotic and necrotic cell death associated with disease pathogenesis (103, 104). Multiple proteins have been proposed to constitute the PTP and thus to play a role in PTP opening by Ca^2+^ or ROS challenge, such as VDAC1 in the OMM, ANT in the IMM, and cyclophilin D in the matrix (105, 106). However, silencing approaches have only confirmed cyclophilin D as being essential for Ca^2+^-sensitive PTP opening. One recent view considered parts of the F_o_F_1_ ATPase as components of the PTP, while other candidates has also emerged [for review, see Ref. (107)]. Recently, SPG7 at the IMM has been proposed as a key component of Ca^2+^- and ROS-induced PTP opening, forming a complex with VDAC1 at the OMM and cyclophilin D in the matrix (108).

A potential candidate for the [Ca^2+^]_m_/H^+^ anti-porter was suggested, the leucine zipper-EF-hand-containing transmembrane protein 1 (Letm1) (19, 92, 109–111). Letm1 has two Ca^2+^-binding EF hand domains and catalyzes the electronic exchange of Ca^2+^ for H^+^. Letm1 Ca^2+^ transport activity is pH-sensitive and is inhibited by RuR (111). Letm1 not only imports Ca^2+^ into the matrix through the IMM but can also extrude Ca^2+^ from the matrix when [Ca^2+^]_m_ concentration is high (19, 109, 110).

Channels function in Ca^2+^ transport in membranes other than in the mitochondria as ryanodine receptors (RyRs) and the transient receptor potential 3 (TRPC3) channel were reported to function in Ca^2+^ homeostasis [for review, see Ref. (112, 113)]; RyRs, the main Ca^2+^-release channels in the SR/ER in excitable cells, were reported to be expressed at the IMM and mediate Ca^2+^ uptake in cardiomyocytes (114, 115). Recently, it was demonstrated that neuronal mitochondria express RyR at the IMM and accumulate Ca^2+^ in a manner that can be inhibited by dantrolene or ryanodine (116). Finally, canonical TRPC3 was shown to be located in the IMM and contributing to [Ca^2+^]_m_ uptake and thus functions in regulating [Ca^2+^]_m_ homeostasis (117).

## VDAC1 at the Nexus of Mitochondria-Mediated Apoptosis

Mitochondria-mediated or intrinsic apoptotic pathway is activated *via* the release of mitochondrial pro-apoptotic proteins (e.g., Cyto *c*, AIF, Smac/DIABLO) from the IMS to the cytosol (32, 33, 52, 54, 118–126), leading to the activation of caspases. Some models for the release of apoptotic proteins suggest that release exclusively involves an increase in OMM permeability due to the formation of a channel large enough to allow the passage of apoptogenic proteins (32, 33, 124, 127–129), while others consider release to be due to disruption of OMM integrity (120, 130, 131). Recent studies demonstrated that upon apoptosis induction, VDAC1 is oligomerized to form a large pore, allowing the release of mitochondrial pro-apoptotic proteins (129, 132–139). VDAC1 oligomerization was found to be a general mechanism common to numerous apoptogens acting *via* different initiating cascades (135, 140, 141). Moreover, apoptosis inhibitors (135, 142) and recently identified VDAC1-interacting molecules [diphenylamine-2-carboxylate (DPC)] (40) and a molecule developed in our lab designated as VBIT-4 were found to prevent VDAC1 oligomerization and subsequent apoptosis (143). Furthermore, cyathin-R, a cyathane-type diterpenoid derived from a fungal secondary metabolite library from the medicinal fungus *Cyathus africanus*, was found to interact with purified VDAC1 and reduce its channel activity, as well as induce apoptosis *via* promoting VDAC1 oligomerization and the associated cytochrome *c* release in Bax/Bak-depleted cells but not when VDAC1 was depleted. Cyathin-R-induced apoptosis was inhibited by DPC (142).

VDAC1 also regulates apoptosis *via* the direct interaction with the anti-apoptotic protein HK (144–152), with apoptosis-regulating proteins, such as Bcl-2, Bcl-xL (33, 47, 48, 50, 144, 153–155), and with the pro-apoptotic proteins Bax and Bak (156).

### Ca^2+^-Induced Apoptosis through VDAC1 Overexpression

Apoptosis induction affects cell Ca^2+^ homeostasis and energy production (157). The intrinsic apoptotic pathway, initiated in response to various stimuli, including high [Ca^2+^]_i_, oxygen radicals, activation of pro-apoptotic Bcl-2 family proteins, UV damage, and various anticancer drugs and cytotoxic agents, such as thapsigargin, staurosporine, As_2_O_3_, and selenite, disrupts cellular Ca^2+^ homeostasis and induces apoptosis (140). Indeed, the contribution of Ca^2+^ signals to cell death is well documented, and a few mechanisms that connect apoptotic stimuli, *via* a rise in [Ca^2+^]_i_, to cell death have been suggested (158–164).

**Figure 2:**
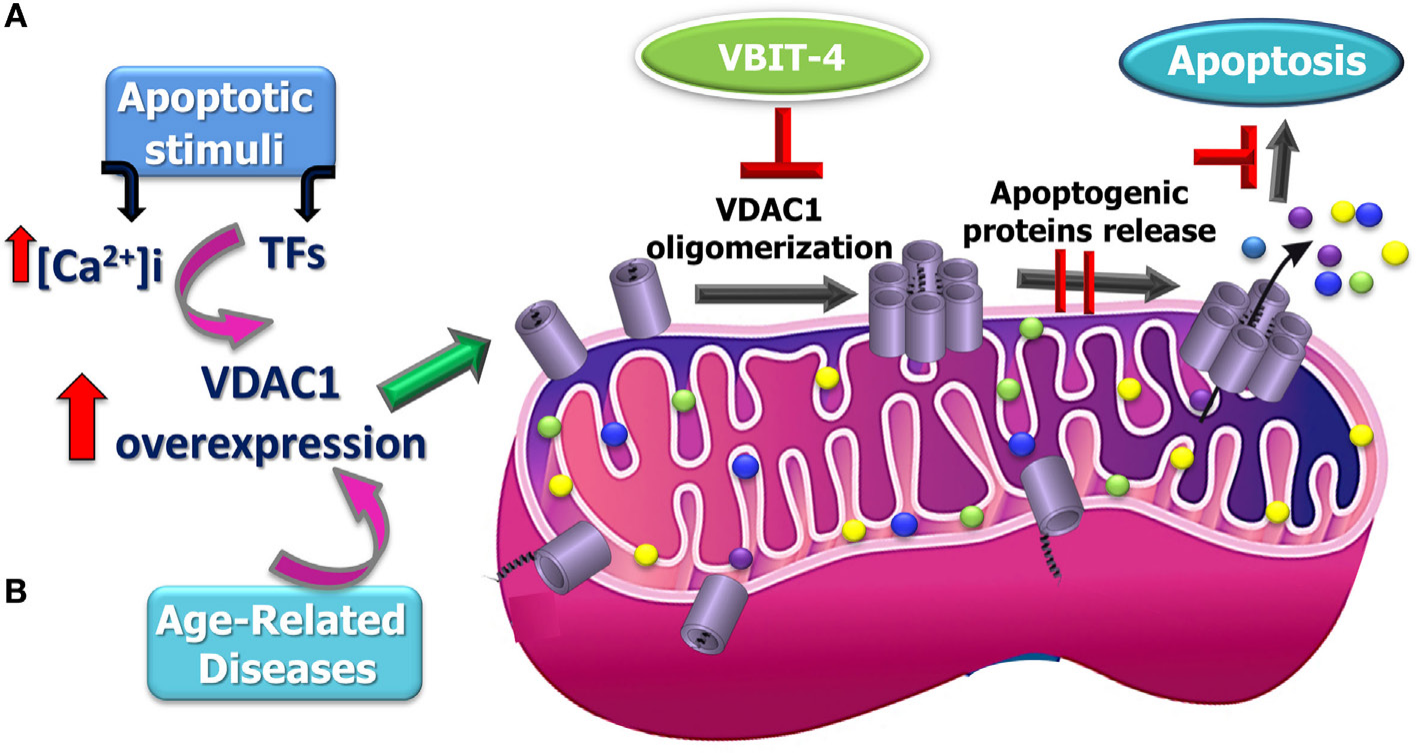
**Proposed model for apoptosis stimuli-induced increase in voltage-dependent anion channel 1 (VDAC1) expression levels leading to VDAC1 oligomerization, Cyto *c* release, and apoptosis and possible inhibition steps**. A schematic model describing the novel pathway proposed for apoptosis induction involving elevation of [Ca^2+^]_i_ leading to VDAC1 overexpression. **(A)** This facilitates VDAC1 oligomerization to form a large channel mediating cytochrome *c* release from the mitochondrion into the cytosol, resulting in apoptosis activation. It is proposed that the overexpression of VDAC1 in diseases such as Alzheimer’s disease, cardiovascular diseases, and diabetes is associated mitochondrial dysfunction, including apoptosis induction **(B)**.

Recently, it was demonstrated that a panel of apoptotic inducers, such as UV irradiation, H_2_O_2_, etoposide, cisplatin, or selenite, elevated [Ca^2+^]_i_ and upregulated VDAC1 expression levels in a Ca^2+^-dependent manner (Table 1), resulting in VDAC1 oligomerization, Cyto *c* release, and subsequent apoptosis (140, 141) (Figure 2). Furthermore, direct elevation of [Ca^2+^]_i_ by the Ca^2+^-mobilizing agents A23187, ionomycin, or thapsigargin led to VDAC1 overexpression, VDAC1 oligomerization, and apoptosis, while decreasing [Ca^2+^]_i_ using the cell-permeable Ca^2+^-chelating reagent BAPTA-AM inhibited these events (141).

**Table 1:** **Anticancer, pro-apoptotic drugs, and chemical agents that increase voltage-dependent anion channel 1 expression level in cancer cells**.

Drugs or chemical agent	Cancer cell type	Reference
*Prednisolone*—synthetic glucocorticoid, a derivative of cortisol, used to treat a variety of inflammatory and autoimmune conditions and some cancers	Acute lymphoblastic leukemia cell lines, REH, 697, Sup-B15, and RS4;11	(165)
*Cisplatin*—a chemotherapy drug, the first member of a class of platinum-containing anticancer drugs	Cervix squamous cell carcinoma line (A431), human cervical adenocarcinoma (HeLa), non-small human lung carcinoma (A549), and human ovarian carcinoma (SKOV3)	(141, 166)
*Mechlorethamine and its derivative, melphalan*—DNA cross-linking agents, a group of anticancer chemotherapeutic drugs	Human cervical adenocarcinoma (HeLa)	(167)
*ROS*—reactive oxygen species (H_2_O_2_ and sodium nitroprusside)	Human cervical adenocarcinoma (HeLa), non-small human lung carcinoma (A549), human ovarian carcinoma (SKOV3), and rat PC12 cells	(141, 168)
*UV irradiation*	B cell mouse lymphoma (LYas)	(169)
*Arbutin*—(hydroquinone-*O*-β-d-glucopyranoside), tyrosinase inhibitor, and potential anticancer agent, extracted from the bearberry plant	Human malignant melanoma cells (A375)	(170, 171)
*Orf3*—hepatitis E virus protein	Hepatoma cells	(172)
*Somatostatin*—a peptide hormone	Human prostate cancer cell line (LNCaP)	(173)
*Endostatin*—20-kDa C-terminal fragment derived from type XVIII collage	Human microvascular endothelial cells	(126)
*Selenite*—inorganic compound	Human cervix carcinoma (HeLa) cells	(141, 174)
*Thapsigargin*—non-competitive inhibitor of the sarco/endoplasmic reticulum Ca^2+^-ATPase, extracted from the plant *Thapsia garganica*	U266 myeloma cells and human cervical adenocarcinoma (HeLa) cells	(141, 175)
*Etoposide*—topoisomerase inhibitor, cytotoxic anticancer drug	Human cervical adenocarcinoma (HeLa), non-small human lung carcinoma (A549), and human ovarian carcinoma (SKOV3)	(141)
*Arsenic trioxide* $\left({\text{As}}_{\text{2}}{\text{O}}_{3}^{-}\right)$—inorganic compound	Human cervical adenocarcinoma (HeLa), non-small human lung carcinoma (A549), and human ovarian carcinoma (SKOV3)	(141)

It has also been shown that the sensitivity of the CD45-positive (CD45^+^) U266 myeloma cell line to various apoptotic stimuli is well correlated with the elevated levels of VDAC1 expression that follow Ca^2+^ signals in response to apoptosis stimulation (169, 175). This suggests that apoptosis-inducing agents act by increasing [Ca^2+^]_i_ and that this, in turn, leads to an upregulation of VDAC1 expression, which is connected to apoptosis induction (Table 1). The proposed sequence of events leading to VDAC1-mediated apoptosis can be schematically depicted as: 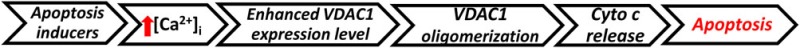


Support for this model comes with the findings that several VDAC-interacting molecules prevent its oligomerization, the elevation in [Ca^2+^]_i_ associated with apoptosis induction, Cyto *c* release, and apoptosis (40, 41, 141, 151, 165, 166, 170, 171, 173, 176–178). DIDS was shown to prevent the apoptosis stimuli-inducing increase in [Ca^2+^]_i_ levels (40) and Ca^2+^-mediated oxidative stress and apoptosis, as induced by 5-aminolevulinic (41). The small molecules AKOS-022 and VBIT-4 that bind to VDAC1 prevent its oligomerization, the elevation [Ca^2+^]_i_ associated with apoptosis induction, Cyto *c* release, and apoptosis (166). Furthermore, mitochondria-mediated apoptosis was correlated with VDAC1 expression levels (141, 165–175). Thus, although different apoptosis inducers elicit cell death *via* different mechanisms, all induce VDAC1 overexpression in a Ca^2+^-dependent manner, raising the possibility that elevating [Ca^2+^]_i_ represents a common mechanism for various apoptosis stimuli, subsequently leading to an elevation in VDAC1 expression. We, therefore, suggest that the upregulation of VDAC1 expression constitutes a major mechanism by which apoptosis inducers lead to apoptosis (Figure 2).

Although many studies in various experimental systems have demonstrated increased VDAC1 expression levels following apoptosis stimulation (Table 1), only a few have linked VDAC1 overexpression to the rise in [Ca^2+^]_i_ following apoptosis induction. Indeed, the expression level of VDAC1 has been shown to be a crucial factor in the process of mitochondria-mediated apoptosis (141, 165–175). Moreover, exogenous VDAC1 expression leads to apoptosis in the absence of any apoptotic stimulus (32, 34, 137, 141, 144, 151, 165, 179). There are several potential Ca^2+^-dependent steps that could contribute to the process of gene expression and a few, such as mRNA transcription, elongation, splicing, stability, and translation, have been suggested as being regulated by Ca^2+^ (180, 181).

This new mode of action for apoptosis stimulus involving increased expression of VDAC1 leading to dynamic VDAC1 oligomerization, release of Cyto *c*, and apoptosis provides a platform for developing a new class of anticancer drugs modulating VDAC1 expression *via* its promoter.

## VDAC1 and Ca^2+^ in Cancer and Other Diseases

Various cancer hallmarks, such as proliferation, migration, angiogenesis, invasion abilities, and resistance to cell death, are associated with alterations of Ca^2+^ homeostasis (2). As a transporter of metabolites and Ca^2+^, VDAC1 contributes to the metabolic phenotype of cancer cells, possibly as reflected in its overexpression in many cancer types (182, 183). Moreover, its downregulation resulted in reduced metabolite exchanges between mitochondria and cytosol and inhibited cell and tumor growth (122, 176, 182, 184, 185).

Tumor cells exhibit a well-developed capacity for modulating [Ca^2+^]_i_ levels by remodeling the cellular machinery that participates in processes that determine Ca^2+^ dynamics and homeostasis, as well as changes in sensitivity to cell death induction (186). It was recently demonstrated that the basal [Ca^2+^]_m_ uptake *via* the ER–mitochondria junction is essential for tumorigenic cell viability, and that inhibition of this pathway in cancer cells might be used as a therapeutic approach (187). Moreover, some cancer cells are addicted to such constitutive Ca^2+^ transfer to sustain their mitochondrial metabolism, particularly nucleoside production (188). Thus, the increase in VDAC1 levels in cancer (182, 183) also contributes to this enhanced transport of Ca^2+^.

In diabetic mouse coronary vascular endothelial cells (MCECs), VDAC levels were increased, as were [Ca^2+^]_m_, mitochondrial O_2_ production, and PTP opening activity (189). Downregulation of VDAC1 in diabetic MCECs decreased [Ca^2+^]_m_ and subsequently normalized the levels of PTP activity and mitochondrial ROS production (190). VDAC1 has proposed to mediate the protective effects of hesperidin, a bioactive flavonoid compound, against amyloid β-induced mitochondrial dysfunction, mitochondrial PTP opening, [Ca^2+^]_i_ increase, and ROS production (191). It has also shown that blocking of VDAC1-mediated [Ca^2+^]_m_ release in Schwann cells prevented demyelinating neuropathies (192). Thus, VDAC function in Ca^2+^ homeostasis is connected to several diseases.

## Author Contributions

VS-B wrote the review; SD and AM helped in writing.

## Conflict of Interest Statement

The authors declare that the research was conducted in the absence of any commercial or financial relationships that could be construed as a potential conflict of interest.

## References

[1] Berridge MJ Bootman MD Roderick HL. Calcium signalling: dynamics, homeostasis and remodelling. Nat Rev Mol Cell Biol (2003) 4:517–29. 10.1038/nrm1155 12838335

[2] Roderick HL Cook SJ. Ca2+ signalling checkpoints in cancer: remodelling Ca2+ for cancer cell proliferation and survival. Nat Rev Cancer (2008) 8:361–75. 10.1038/nrc2374 18432251

[3] Berridge MJ Lipp P Bootman MD. The versatility and universality of calcium signalling. Nat Rev Mol Cell Biol (2000) 1:11–21. 10.1038/35036191 11413485

[4] Rizzuto R De Stefani D Raffaello A Mammucari C. Mitochondria as sensors and regulators of calcium signalling. Nat Rev Mol Cell Biol (2012) 13:566–78. 10.1038/nrm3412 22850819

[5] Cox DA Matlib MA. A role for the mitochondrial Na(+)-Ca2+ exchanger in the regulation of oxidative phosphorylation in isolated heart mitochondria. J Biol Chem (1993) 268:938–47. 8419373

[6] Glancy B Balaban RS. Role of mitochondrial Ca2+ in the regulation of cellular energetics. Biochemistry (2012) 51:2959–73. 10.1021/bi2018909 22443365 PMC3332087

[7] Nicholls DG. Mitochondria and calcium signaling. Cell Calcium (2005) 38:311–7. 10.1016/j.ceca.2005.06.011 16087232

[8] Gunter TE Buntinas L Sparagna G Eliseev R Gunter K. Mitochondrial calcium transport: mechanisms and functions. Cell Calcium (2000) 28:285–96. 10.1054/ceca.2000.0168 11115368

[9] Xia H Mao Q Eliason SL Harper SQ Martins IH Orr HT RNAi suppresses polyglutamine-induced neurodegeneration in a model of spinocerebellar ataxia. Nat Med (2004) 10:816–20. 10.1038/nm1076 15235598

[10] Giacomello M Drago I Pizzo P Pozzan T. Mitochondrial Ca2+ as a key regulator of cell life and death. Cell Death Differ (2007) 14:1267–74. 10.1038/sj.cdd.4402147 17431419

[11] Maechler P Kennedy ED Pozzan T Wollheim CB. Mitochondrial activation directly triggers the exocytosis of insulin in permeabilized pancreatic beta-cells. EMBO J (1997) 16:3833–41. 10.1093/emboj/16.13.3833 9233793 PMC1170007

[12] Lee B Miles PD Vargas L Luan P Glasco S Kushnareva Y Inhibition of mitochondrial Na+-Ca2+ exchanger increases mitochondrial metabolism and potentiates glucose-stimulated insulin secretion in rat pancreatic islets. Diabetes (2003) 52:965–73. 10.2337/diabetes.52.4.965 12663468

[13] Prins D Michalak M Organellar calcium buffers. Cold Spring Harb Perspect Biol (2011) 3:1–16. 10.1101/cshperspect.a004069 PMC3039927 21421925

[14] Denton RM. Regulation of mitochondrial dehydrogenases by calcium ions. Biochim Biophys Acta (2009) 1787:1309–16. 10.1016/j.bbabio.2009.01.005 19413950

[15] Nichols BJ Denton RM. Towards the molecular basis for the regulation of mitochondrial dehydrogenases by calcium ions. Mol Cell Biochem (1995) 149:203–12. 10.1007/BF01076578 8569730

[16] Cardenas C Miller RA Smith I Bui T Molgo J Muller M Essential regulation of cell bioenergetics by constitutive InsP3 receptor Ca2+ transfer to mitochondria. Cell (2010) 142:270–83. 10.1016/j.cell.2010.06.007 20655468 PMC2911450

[17] Rizzuto R Bernardi P Pozzan T. Mitochondria as all-round players of the calcium game. J Physiol (2000) 529:37–47. 10.1111/j.1469-7793.2000.00037.x 11080249 PMC2270183

[18] De Stefani D Rizzuto R Pozzan T. Enjoy the trip: calcium in mitochondria back and forth. Annu Rev Biochem (2016) 85:161–92. 10.1146/annurev-biochem-060614-034216 27145841

[19] Santo-Domingo J Demaurex N. Calcium uptake mechanisms of mitochondria. Biochim Biophys Acta (2010) 1797:907–12. 10.1016/j.bbabio.2010.01.005 20079335

[20] Sekler I Standing of giants shoulders the story of the mitochondrial Na+Ca2+ exchanger. Biochem Biophys Res Commun (2015) 460:50–2. 10.1016/j.bbrc.2015.02.170 25998733

[21] Takeuchi A Kim B Matsuoka S. The destiny of Ca(2+) released by mitochondria. J Physiol Sci (2015) 65:11–24. 10.1007/s12576-014-0326-7 24994533 PMC4276810

[22] Nita LI Hershfinkel M Sekler I. Life after the birth of the mitochondrial Na+/Ca2+ exchanger, NCLX. Sci China Life Sci (2015) 58:59–65. 10.1007/s11427-014-4789-9 25576453

[23] Baughman JM Perocchi F Girgis HS Plovanich M Belcher-Timme CA Sancak Y Integrative genomics identifies MCU as an essential component of the mitochondrial calcium uniporter. Nature (2011) 476:341–5. 10.1038/nature10234 21685886 PMC3486726

[24] De Stefani D Raffaello A Teardo E Szabo I Rizzuto R. A forty-kilodalton protein of the inner membrane is the mitochondrial calcium uniporter. Nature (2011) 476:336–40. 10.1038/nature10230 21685888 PMC4141877

[25] Palty R Silverman WF Hershfinkel M Caporale T Sensi SL Parnis J NCLX is an essential component of mitochondrial Na+/Ca2+ exchange. Proc Natl Acad Sci U S A (2010) 107:436–41. 10.1073/pnas.0908099107 20018762 PMC2806722

[26] Boyman L Williams GS Khananshvili D Sekler I Lederer WJ NCLX: the mitochondrial sodium calcium exchanger. J Mol Cell Cardiol (2013) 59:205–13. 10.1016/j.yjmcc.2013.03.012 23538132 PMC3951392

[27] Gincel D Zaid H Shoshan-Barmatz V. Calcium binding and translocation by the voltage-dependent anion channel: a possible regulatory mechanism in mitochondrial function. Biochem J (2001) 358:147–55. 10.1042/bj3580147 11485562 PMC1222042

[28] Rapizzi E Pinton P Szabadkai G Wieckowski MR Vandecasteele G Baird G Recombinant expression of the voltage-dependent anion channel enhances the transfer of Ca2+ microdomains to mitochondria. J Cell Biol (2002) 159:613–24. 10.1083/jcb.200205091 12438411 PMC2173108

[29] Tan W Colombini M. VDAC closure increases calcium ion flux. Biochim Biophys Acta (2007) 1768:2510–5. 10.1016/j.bbamem.2007.06.002 17617374 PMC2220155

[30] Bathori G Csordas G Garcia-Perez C Davies E Hajnoczky G Ca2+-dependent control of the permeability properties of the mitochondrial outer membrane and voltage-dependent anion-selective channel (VDAC). J Biol Chem (2006) 281:17347–58. 10.1074/jbc.M600906200 16597621

[31] De Pinto V Guarino F Guarnera A Messina A Reina S Tomasello FM Characterization of human VDAC isoforms: a peculiar function for VDAC3? Biochim Biophys Acta (2010) 1797:1268–75. 10.1016/j.bbabio.2010.01.031 20138821

[32] Shoshan-Barmatz V Ben-Hail D. VDAC, a multi-functional mitochondrial protein as a pharmacological target. Mitochondrion (2012) 12:24–34. 10.1016/j.mito.2011.04.001 21530686

[33] Shoshan-Barmatz V De Pinto V Zweckstetter M Raviv Z Keinan N Arbel N. VDAC, a multi-functional mitochondrial protein regulating cell life and death. Mol Aspects Med (2010) 31:227–85. 10.1016/j.mam.2010.03.002 20346371

[34] Shoshan-Barmatz V Mizrachi D. VDAC1: from structure to cancer therapy. Front Oncol (2012) 2:164. 10.3389/fonc.2012.00164 23233904 PMC3516065

[35] Dolder M Wendt S Wallimann T. Mitochondrial creatine kinase in contact sites: interaction with porin and adenine nucleotide translocase, role in permeability transition and sensitivity to oxidative damage. Biol Signals Recept (2001) 10:93–111. 10.1159/000046878 11223643

[36] Wu W Zhao S. Metabolic changes in cancer: beyond the Warburg effect. Acta Biochim Biophys Sin (Shanghai) (2013) 45:18–26. 10.1093/abbs/gms104 23257292

[37] Madesh M Hajnoczky G. VDAC-dependent permeabilization of the outer mitochondrial membrane by superoxide induces rapid and massive cytochrome c release. J Cell Biol (2001) 155:1003–15. 10.1083/jcb.200105057 11739410 PMC2150912

[38] De Stefani D Bononi A Romagnoli A Messina A De Pinto V Pinton P VDAC1 selectively transfers apoptotic Ca2+ signals to mitochondria. Cell Death Differ (2012) 19:267–73. 10.1038/cdd.2011.92 21720385 PMC3263501

[39] Huang H Hu X Eno CO Zhao G Li C White C. An interaction between Bcl-xL and the voltage-dependent anion channel (VDAC) promotes mitochondrial Ca2+ uptake. J Biol Chem (2013) 288:19870–81. 10.1074/jbc.M112.448290 23720737 PMC3707689

[40] Ben-Hail D Shoshan-Barmatz V. VDAC1-interacting anion transport inhibitors inhibit VDAC1 oligomerization and apoptosis. Biochim Biophys Acta (2016) 1863:1612–23. 10.1016/j.bbamcr.2016.04.002 27064145

[41] Chen H Gao W Yang Y Guo S Wang H Wang W Inhibition of VDAC1 prevents Ca(2)(+)-mediated oxidative stress and apoptosis induced by 5-aminolevulinic acid mediated sonodynamic therapy in THP-1 macrophages. Apoptosis (2014) 19:1712–26. 10.1007/s10495-014-1045-5 25342393

[42] Charuk JH Pirraglia CA Reithmeier RA. Interaction of ruthenium red with Ca2(+)-binding proteins. Anal Biochem (1990) 188:123–31. 10.1016/0003-2697(90)90539-L 1699445

[43] Israelson A Abu-Hamad S Zaid H Nahon E Shoshan-Barmatz V. Localization of the voltage-dependent anion channel-1 Ca2+-binding sites. Cell Calcium (2007) 41:235–44. 10.1016/j.ceca.2006.06.005 16930689

[44] Gincel D Vardi N Shoshan-Barmatz V. Retinal voltage-dependent anion channel: characterization and cellular localization. Invest Ophthalmol Vis Sci (2002) 43:2097–104. 12091402

[45] Israelson A Arzoine L Abu-hamad S Khodorkovsky V Shoshan-Barmatz V. A photoactivable probe for calcium binding proteins. Chem Biol (2005) 12:1169–78. 10.1016/j.chembiol.2005.08.006 16298296

[46] Israelson A Zaid H Abu-Hamad S Nahon E Shoshan-Barmatz V. Mapping the ruthenium red-binding site of the voltage-dependent anion channel-1. Cell Calcium (2008) 43:196–204. 10.1016/j.ceca.2007.05.006 17590433

[47] Arbel N Ben-Hail D Shoshan-Barmatz V. Mediation of the antiapoptotic activity of Bcl-xL protein upon interaction with VDAC1 protein. J Biol Chem (2012) 287:23152–61. 10.1074/jbc.M112.345918 22589539 PMC3391160

[48] Arbel N Shoshan-Barmatz V. Voltage-dependent anion channel 1-based peptides interact with Bcl-2 to prevent antiapoptotic activity. J Biol Chem (2010) 285:6053–62. 10.1074/jbc.M109.082990 20037155 PMC2825399

[49] Kholmukhamedov EL Czerny C Lovelace G Beeson KC Baker T Johnson CB [The role of the voltage-dependent anion channels in the outer membrane of mitochondria in the regulation of cellular metabolism]. Biofizika (2010) 55:822–33. 21033348 PMC4547860

[50] Malia TJ Wagner G. NMR structural investigation of the mitochondrial outer membrane protein VDAC and its interaction with antiapoptotic Bcl-xL. Biochemistry (2007) 46:514–25. 10.1021/bi061577h 17209561 PMC2579276

[51] Monaco G Decrock E Arbel N van Vliet AR La Rovere RM De Smedt H The BH4 domain of anti-apoptotic Bcl-XL, but not that of the related Bcl-2, limits the voltage-dependent anion channel 1 (VDAC1)-mediated transfer of pro-apoptotic Ca2+ signals to mitochondria. J Biol Chem (2015) 290:9150–61. 10.1074/jbc.M114.622514 25681439 PMC4423701

[52] Shimizu S Konishi A Kodama T Tsujimoto Y. BH4 domain of antiapoptotic Bcl-2 family members closes voltage-dependent anion channel and inhibits apoptotic mitochondrial changes and cell death. Proc Natl Acad Sci U S A (2000) 97:3100–5. 10.1073/pnas.97.7.3100 10737788 PMC16199

[53] Sugiyama T Shimizu S Matsuoka Y Yoneda Y Tsujimoto Y. Activation of mitochondrial voltage-dependent anion channel by a pro-apoptotic BH3-only protein Bim. Oncogene (2002) 21:4944–56. 10.1038/sj.onc.1205621 12118373

[54] Tajeddine N Galluzzi L Kepp O Hangen E Morselli E Senovilla L Hierarchical involvement of Bak, VDAC1 and Bax in cisplatin-induced cell death. Oncogene (2008) 27:4221–32. 10.1038/onc.2008.63 18362892

[55] Tsujimoto Y. Cell death regulation by the Bcl-2 protein family in the mitochondria. J Cell Physiol (2003) 195:158–67. 10.1002/jcp.10254 12652643

[56] Hein MY Hubner NC Poser I Cox J Nagaraj N Toyoda Y A human interactome in three quantitative dimensions organized by stoichiometries and abundances. Cell (2015) 163:712–23. 10.1016/j.cell.2015.09.053 26496610

[57] Lv BF Yu CF Chen YY Lu Y Guo JH Song QS Protein tyrosine phosphatase interacting protein 51 (PTPIP51) is a novel mitochondria protein with an N-terminal mitochondrial targeting sequence and induces apoptosis. Apoptosis (2006) 11:1489–501. 10.1007/s10495-006-8882-9 16820967

[58] De Vos KJ Mórotz GM Stoica R Tudor EL Lau K-F Ackerley S VAPB interacts with the mitochondrial protein PTPIP51 to regulate calcium homeostasis. Hum Mol Genet (2012) 21:1299–311. 10.1093/hmg/ddr559 22131369 PMC3284118

[59] Sun J Liao JK. Functional interaction of endothelial nitric oxide synthase with a voltage-dependent anion channel. Proc Natl Acad Sci U S A (2002) 99:13108–13. 10.1073/pnas.202260999 12228731 PMC130594

[60] Viola HM Adams AM Davies SM Fletcher S Filipovska A Hool LC. Impaired functional communication between the L-type calcium channel and mitochondria contributes to metabolic inhibition in the mdx heart. Proc Natl Acad Sci U S A (2014) 111:E2905–14. 10.1073/pnas.1402544111 24969422 PMC4104855

[61] Shoshan-Barmatz V Israelson A. The voltage-dependent anion channel in endoplasmic/sarcoplasmic reticulum: characterization, modulation and possible function. J Membr Biol (2005) 204:57–66. 10.1007/s00232-005-0749-4 16151701

[62] Shoshan-Barmatz V Zalk R Gincel D Vardi N. Subcellular localization of VDAC in mitochondria and ER in the cerebellum. Biochim Biophys Acta (2004) 1657:105–14. 10.1016/j.bbabio.2004.02.009 15238267

[63] Csordás G Renken C Várnai P Walter L Weaver D Buttle KF Structural and functional features and significance of the physical linkage between ER and mitochondria. J Cell Biol (2006) 174:915–21. 10.1083/jcb.200604016 16982799 PMC2064383

[64] Marchi S Patergnani S Pinton P. The endoplasmic reticulum-mitochondria connection: one touch, multiple functions. Biochim Biophys Acta (2014) 1837:461–9. 10.1016/j.bbabio.2013.10.015 24211533

[65] Booth DM Joseph SK Hajnoczky G. Subcellular ROS imaging methods: relevance for the study of calcium signaling. Cell Calcium (2016) 60:65–73. 10.1016/j.ceca.2016.05.001 27209367 PMC4996722

[66] Bernardi P. Mitochondrial transport of cations: channels, exchangers, and permeability transition. Physiol Rev (1999) 79:1127–55. 10508231 10.1152/physrev.1999.79.4.1127

[67] Foskett JK Philipson B The mitochondrial Ca2 + uniporter complex. J Mol Cell Cardiol (2015) 78:3–8. 10.1016/j.yjmcc.2014.11.015 25463276 PMC4307384

[68] Vasington FD Gazzotti P Tiozzo R Carafoli E The effect of ruthenium red on Ca 2+ transport and respiration in rat liver mitochondria. Biochim Biophys Acta (1972) 256:43–54. 10.1016/0005-2728(72)90161-2 4257941

[69] Kamer KJ Sancak Y Mootha VK The uniporter: from newly identified parts to function. Biochem Biophys Res Commun (2014) 449:370–2. 10.1016/j.bbrc.2014.04.143 24814702

[70] Marchi S Pinton P. The mitochondrial calcium uniporter complex: molecular components, structure and physiopathological implications. J Physiol (2014) 592:829–39. 10.1113/jphysiol.2013.268235 24366263 PMC3948548

[71] Lee Y Min CK Kim TG Song HK Lim Y Kim D Structure and function of the N-terminal domain of the human mitochondrial calcium uniporter. EMBO Rep (2015) 16:1318–33. 10.15252/embr.201540436 26341627 PMC4662854

[72] Csordás G Golenár T Seifert EL Kamer KJ Sancak Y Perocchi F MICU1 controls both the threshold and cooperative activation of the mitochondrial Ca(2+) uniporter. Cell Metab (2013) 17:976–87. 10.1016/j.cmet.2013.04.020 23747253 PMC3722067

[73] Hoffman NE Chandramoorthy HC Shanmughapriya S Zhang XQ Vallem S Doonan PJ SLC25A23 augments mitochondrial Ca^2+^ uptake, interacts with MCU, and induces oxidative stress-mediated cell death. Mol Biol Cell (2014) 25:936–47. 10.1091/mbc.E13-08-0502 24430870 PMC3952861

[74] Mallilankaraman K Doonan P Cárdenas C Chandramoorthy HC Müller M Miller R MICU1 is an essential gatekeeper for MCU-mediated mitochondrial Ca2+ uptake that regulates cell survival. Cell (2012) 151:630–44. 10.1016/j.cell.2012.10.011 23101630 PMC3486697

[75] Plovanich M Bogorad RL Sancak Y Kamer KJ Strittmatter L Li AA MICU2, a paralog of MICU1, resides within the mitochondrial uniporter complex to regulate calcium handling. PLoS One (2013) 8:e55785. 10.1371/journal.pone.0055785 23409044 PMC3567112

[76] Sancak Y Markhard AL Kitami T Kovács-Bogdán E Kamer KJ Udeshi ND EMRE is an essential component of the mitochondrial calcium uniporter complex. Science (2013) 342:1379–82. 10.1126/science.1242993 24231807 PMC4091629

[77] Paupe V Prudent J Dassa EP Rendon OZ Shoubridge EA. CCDC90A (MCUR1) is a cytochrome c oxidase assembly factor and not a regulator of the mitochondrial calcium uniporter. Cell Metab (2015) 21:109–16. 10.1016/j.cmet.2014.12.004 25565209

[78] Pozzan T Magalhaes P Rizzuto R. The comeback of mitochondria to calcium signalling. Cell Calcium (2000) 28:279–83. 10.1054/ceca.2000.0166 11115367

[79] Xu S Chisholm AD. *C. elegans* epidermal wounding induces a mitochondrial ROS burst that promotes wound repair. Dev Cell (2014) 31:48–60. 10.1016/j.devcel.2014.08.002 25313960 PMC4197410

[80] Alam MR Groschner LN Parichatikanond W Kuo L Bondarenko AI Rost R Mitochondrial Ca^2+^ uptake 1 (MICU1) and mitochondrial Ca^2+^ uniporter (MCU) contribute to metabolism-secretion coupling in clonal pancreatic β-cells. J Biol Chem (2012) 287:34445–54. 10.1074/jbc.M112.392084 22904319 PMC3464549

[81] Drago I De Stefani D Rizzuto R Pozzan T Mitochondrial Ca2+ uptake contributes to buffering cytoplasmic Ca2+ peaks in cardiomyocytes. Proc Natl Acad Sci U S A (2012) 109:12986–91. 10.1073/pnas.1210718109 22822213 PMC3420165

[82] Pan X Liu J Nguyen T Liu C Sun J Teng Y The physiological role of mitochondrial calcium revealed by mice lacking the mitochondrial calcium uniporter. Nat Cell Biol (2013) 15:1464–72. 10.1038/ncb2868 24212091 PMC3852190

[83] Qiu J Tan Y-W Hagenston AM Martel M-A Kneisel N Skehel PA Mitochondrial calcium uniporter MCU controls excitotoxicity and is transcriptionally repressed by neuroprotective nuclear calcium signals. Nat Commun (2013) 4:2034. 10.1038/ncomms3034 23774321 PMC3709514

[84] Quan X Nguyen TT Choi S-K Xu S Das R Cha S-K Essential role of mitochondrial Ca2+ uniporter in the generation of mitochondrial pH gradient and metabolism-secretion coupling in insulin-releasing cells. J Biol Chem (2015) 290:4086–96. 10.1074/jbc.M114.632547 25548283 PMC4326818

[85] Rasmussen TP Wu Y Joiner ML Koval OM Wilson NR Luczak ED Inhibition of MCU forces extramitochondrial adaptations governing physiological and pathological stress responses in heart. Proc Natl Acad Sci U S A (2015) 112:9129–34. 10.1073/pnas.1504705112 26153425 PMC4517214

[86] Holmström KM Pan X Liu JC Menazza S Liu J Nguyen TT Assessment of cardiac function in mice lacking the mitochondrial calcium uniporter. J Mol Cell Cardiol (2015) 85:178–82. 10.1016/j.yjmcc.2015.05.022 26057074 PMC4530042

[87] Luongo TS Lambert JP Yuan A Zhang X Gross P Song J The mitochondrial calcium uniporter matches energetic supply with cardiac workload during stress and modulates permeability transition. Cell Rep (2015) 12:23–34. 10.1016/j.celrep.2015.06.017 26119731 PMC4517182

[88] Murphy E Pan X Nguyen T Liu J Holmstrom KM Finkel T. Unresolved questions from the analysis of mice lacking MCU expression. Biochem Biophys Res Commun (2014) 449:384–5. 10.1016/j.bbrc.2014.04.144 24792186 PMC4214067

[89] Kwong JQ Lu X Correll RN Schwanekamp JA Vagnozzi RJ Sargent MA The mitochondrial calcium uniporter selectively matches metabolic output to acute contractile stress in the heart. Cell Rep (2015) 12:15–22. 10.1016/j.celrep.2015.06.002 26119742 PMC4497842

[90] Murphy E Eisner DA Regulation of intracellular and mitochondrial Na in health and disease. Circ Res (2009) 104:292–303. 10.1161/CIRCRESAHA.108.189050 19213964 PMC2662399

[91] Kim B Takeuchi A Hikida M Matsuoka S. Roles of the mitochondrial Na(+)-Ca(2+) exchanger, NCLX, in B lymphocyte chemotaxis. Sci Rep (2016) 6:28378. 10.1038/srep28378 27328625 PMC4916421

[92] Jiang D Zhao L Clish CB Clapham DE. Letm1, the mitochondrial Ca2+/H+ antiporter, is essential for normal glucose metabolism and alters brain function in Wolf-Hirschhorn syndrome. Proc Natl Acad Sci U S A (2013) 110:E2249–54. 10.1073/pnas.1308558110 23716663 PMC3683736

[93] Palty R Hershfinkel M Sekler I. Molecular identity and functional properties of the mitochondrial Na+/Ca2+ exchanger. J Biol Chem (2012) 287:31650–7. 10.1074/jbc.R112.355867 22822063 PMC3442499

[94] Arco AD Satrústegui J New mitochondrial carriers: an overview. Cell Mol Life Sci (2005) 62:2204–27. 10.1007/s00018-005-5197-x 16132231 PMC11139166

[95] McCormack JG Denton RM. Mitochondrial Ca2+ transport and the role of intramitochondrial Ca2+ in the regulation of energy metabolism. Dev Neurosci (1993) 15:165–73. 10.1159/000111332 7805568

[96] Palty R Ohana E Hershfinkel M Volokita M Elgazar V Beharier O Lithium-calcium exchange is mediated by a distinct potassium-independent sodium-calcium exchanger. J Biol Chem (2004) 279:25234–40. 10.1074/jbc.M401229200 15060069

[97] De Marchi U Santo-Domingo J Castelbou C Sekler I Wiederkehr A Demaurex N. NCLX protein, but not LETM1, mediates mitochondrial Ca2+ extrusion, thereby limiting Ca2+-induced NAD(P)H production and modulating matrix redox state. J Biol Chem (2014) 289:20377–85. 10.1074/jbc.M113.540898 24898248 PMC4106350

[98] Williams GS Boyman L Lederer WJ. Mitochondrial calcium and the regulation of metabolism in the heart. J Mol Cell Cardiol (2015) 78:35–45. 10.1016/j.yjmcc.2014.10.019 25450609 PMC6534814

[99] Takeuchi A Kim B Matsuoka S. The mitochondrial Na+-Ca2+ exchanger, NCLX, regulates automaticity of HL-1 cardiomyocytes. Sci Rep (2013) 3:2766. 10.1038/srep02766 24067497 PMC3783885

[100] Murphy E Cross H Steenbergen C Sodium regulation during ischemia versus reperfusion and its role in injury. Circ Res (1999) 84:1469–70. 10.1161/01.RES.84.12.1469 10381900

[101] Babsky A Doliba N Doliba N Savchenko A Wehrli S Osbakken M. Na+ effects on mitochondrial respiration and oxidative phosphorylation in diabetic hearts. Exp Biol Med (2001) 226:543–51. 11395924 10.1177/153537020122600606

[102] Kostic M Ludtmann MH Bading H Hershfinkel M Steer E Chu CT PKA phosphorylation of NCLX reverses mitochondrial calcium overload and depolarization, promoting survival of PINK1-deficient dopaminergic neurons. Cell Rep (2015) 13:376–86. 10.1016/j.celrep.2015.08.079 26440884 PMC4709126

[103] Tsujimoto Y Shimizu S. Role of the mitochondrial membrane permeability transition in cell death. Apoptosis (2007) 12:835–40. 10.1007/s10495-006-0525-7 17136322

[104] Rasola A Bernardi P Mitochondrial permeability transition in Ca2+-dependent apoptosis and necrosis. Cell Calcium (2011) 50:222–33. 10.1016/j.ceca.2011.04.007 21601280

[105] Elrod JW Wong R Mishra S Vagnozzi RJ Sakthievel B Goonasekera SA Cyclophilin D controls mitochondrial pore-dependent Ca(2+) exchange, metabolic flexibility, and propensity for heart failure in mice. J Clin Invest (2010) 120:3680–7. 10.1172/JCI43171 20890047 PMC2947235

[106] Galluzzi L Kroemer G Mitochondrial apoptosis without VDAC. Nat Cell Biol (2007) 9:487–9. 10.1038/ncb0507-487 17473857

[107] Biasutto L Azzolini M Szabò I Zoratti M. The mitochondrial permeability transition pore in AD 2016: an update. Biochim Biophys Acta (2016) 1863:2515–30. 10.1016/j.bbamcr.2016.02.012 26902508

[108] Shanmughapriya S Rajan S Hoffman NE Higgins AM Tomar D Nemani N SPG7 is an essential and conserved component of the mitochondrial permeability transition pore. Mol Cell (2015) 60:47–62. 10.1016/j.molcel.2015.08.009 26387735 PMC4592475

[109] Doonan PJ Chandramoorthy HC Hoffman NE Zhang X Cardenas C Shanmughapriya S LETM1-dependent mitochondrial Ca2+ flux modulates cellular bioenergetics and proliferation. FASEB J (2014) 28:4936–49. 10.1096/fj.14-256453 25077561 PMC4200331

[110] Jiang D Zhao L Clapham DE. Genome-wide RNAi screen identifies Letm1 as a mitochondrial Ca2+/H+ antiporter. Science (2009) 326:144–7. 10.1126/science.1175145 19797662 PMC4067766

[111] Mailloux RJ Harper M-E. Uncoupling proteins and the control of mitochondrial reactive oxygen species production. Free Radic Biol Med (2011) 51:1106–15. 10.1016/j.freeradbiomed.2011.06.022 21762777

[112] Hoppe UC. Mitochondrial calcium channels. FEBS Lett (2010) 584:1975–81. 10.1016/j.febslet.2010.04.017 20388514

[113] Wagner S De Bortoli S Schwarzlander M Szabo I. Regulation of mitochondrial calcium in plants versus animals. J Exp Bot (2016) 67:3809–29. 10.1093/jxb/erw100 27001920

[114] Beutner G Sharma VK Giovannucci DR Yule DI Sheu SS Identification of a ryanodine receptor in rat heart mitochondria. J Biol Chem (2001) 276:21482–8. 10.1074/jbc.M101486200 11297554

[115] Beutner G Sharma VK Lin L Ryu SY Dirksen RT Sheu SS. Type 1 ryanodine receptor in cardiac mitochondria: transducer of excitation-metabolism coupling. Biochim Biophys Acta (2005) 1717:1–10. 10.1016/j.bbamem.2005.09.016 16246297

[116] Jakob R Beutner G Sharma VK Duan Y Gross RA Hurst S Molecular and functional identification of a mitochondrial ryanodine receptor in neurons. Neurosci Lett (2014) 575:7–12. 10.1016/j.neulet.2014.05.026 24861510 PMC4122666

[117] Feng S Li H Tai Y Huang J Su Y Abramowitz J Canonical transient receptor potential 3 channels regulate mitochondrial calcium uptake. Proc Natl Acad Sci U S A (2013) 110:11011–6. 10.1073/pnas.1309531110 23776229 PMC3704010

[118] De Pinto V Messina A Accardi R Aiello R Guarino F Tomasello MF New functions of an old protein: the eukaryotic porin or voltage dependent anion selective channel (VDAC). Ital J Biochem (2003) 52:17–24. 12833633

[119] Granville DJ Gottlieb RA. The mitochondrial voltage-dependent anion channel (VDAC) as a therapeutic target for initiating cell death. Curr Med Chem (2003) 10:1527–33. 10.2174/0929867033457214 12871124

[120] Halestrap AP McStay GP Clarke SJ. The permeability transition pore complex: another view. Biochimie (2002) 84:153–66. 10.1016/S0300-9084(02)01375-5 12022946

[121] Shoshan-Barmatz V Gincel D. The voltage-dependent anion channel: characterization, modulation, and role in mitochondrial function in cell life and death. Cell Biochem Biophys (2003) 39:279–92. 10.1385/CBB:39:3:279 14716081

[122] Shoshan-Barmatz V Golan M. Mitochondrial VDAC1: function in cell life and death and a target for cancer therapy. Curr Med Chem (2012) 19:714–35. 10.2174/092986712798992110 22204343

[123] Shoshan-Barmatz V Israelson A Brdiczka D Sheu SS. The voltage-dependent anion channel (VDAC): function in intracellular signalling, cell life and cell death. Curr Pharm Des (2006) 12:2249–70. 10.2174/138161206777585111 16787253

[124] Tsujimoto Y Shimizu S. The voltage-dependent anion channel: an essential player in apoptosis. Biochimie (2002) 84:187–93. 10.1016/S0300-9084(02)01370-6 12022949

[125] Vyssokikh MY Brdiczka D. The function of complexes between the outer mitochondrial membrane pore (VDAC) and the adenine nucleotide translocase in regulation of energy metabolism and apoptosis. Acta Biochim Pol (2003) 50:389–404. 12833165

[126] Yuan S Fu Y Wang X Shi H Huang Y Song X Voltage-dependent anion channel 1 is involved in endostatin-induced endothelial cell apoptosis. FASEB J (2008) 22:2809–20. 10.1096/fj.08-107417 18381814

[127] Doran E Halestrap AP Cytochrome c release from isolated rat liver mitochondria can occur independently of outer-membrane rupture: possible role of contact sites. Biochem J (2000) 348 Pt 2:343–50. 10.1042/bj3480343 10816428 PMC1221072

[128] Martinou JC Desagher S Antonsson B. Cytochrome c release from mitochondria: all or nothing. Nat Cell Biol (2000) 2:E41–3. 10.1038/35004069 10707095

[129] Zalk R Israelson A Garty ES Azoulay-Zohar H Shoshan-Barmatz V. Oligomeric states of the voltage-dependent anion channel and cytochrome c release from mitochondria. Biochem J (2005) 386:73–83. 10.1042/BJ20041356 15456403 PMC1134768

[130] Bernardi P. The permeability transition pore. Control points of a cyclosporin A-sensitive mitochondrial channel involved in cell death. Biochim Biophys Acta (1996) 1275:5–9. 10.1016/0005-2728(96)00041-2 8688451

[131] Crompton M. The mitochondrial permeability transition pore and its role in cell death. Biochem J (1999) 341(Pt 2):233–49. 10.1042/bj3410233 10393078 PMC1220352

[132] Betaneli V Petrov EP Schwille P. The role of lipids in VDAC oligomerization. Biophys J (2012) 102:523–31. 10.1016/j.bpj.2011.12.049 22325275 PMC3274789

[133] Goncalves RP Buzhynskyy N Prima V Sturgis JN Scheuring S. Supramolecular assembly of VDAC in native mitochondrial outer membranes. J Mol Biol (2007) 369:413–8. 10.1016/j.jmb.2007.03.063 17439818

[134] Hoogenboom BW Suda K Engel A Fotiadis D. The supramolecular assemblies of voltage-dependent anion channels in the native membrane. J Mol Biol (2007) 370:246–55. 10.1016/j.jmb.2007.04.073 17524423

[135] Keinan N Tyomkin D Shoshan-Barmatz V. Oligomerization of the mitochondrial protein voltage-dependent anion channel is coupled to the induction of apoptosis. Mol Cell Biol (2010) 30:5698–709. 10.1128/MCB.00165-10 20937774 PMC3004265

[136] Shoshan-Barmatz V Arbel N Arzoine L VDAC, the voltage-dependent anion channel: function, regulation & mitochondrial signaling in cell life and death. Cell Sci (2008) 4:74–118.

[137] Shoshan-Barmatz V Keinan N Zaid H. Uncovering the role of VDAC in the regulation of cell life and death. J Bioenerg Biomembr (2008) 40:183–91. 10.1007/s10863-008-9147-9 18651212

[138] Shoshan-Barmatz V Mizrachi D Keinan N. Oligomerization of the mitochondrial protein VDAC1: from structure to function and cancer therapy. Prog Mol Biol Transl Sci (2013) 117:303–34. 10.1016/B978-0-12-386931-9.00011-8 23663973

[139] Ujwal R Cascio D Chaptal V Ping P Abramson J. Crystal packing analysis of murine VDAC1 crystals in a lipidic environment reveals novel insights on oligomerization and orientation. Channels (Austin) (2009) 3:167–70. 10.4161/chan.3.3.9196 19574737 PMC3719987

[140] Keinan N Pahima H Ben-Hail D Shoshan-Barmatz V. The role of calcium in VDAC1 oligomerization and mitochondria-mediated apoptosis. Biochim Biophys Acta (2013) 1833:1745–54. 10.1016/j.bbamcr.2013.03.017 23542128

[141] Weisthal S Keinan N Ben-Hail D Arif T Shoshan-Barmatz V. Ca(2+)-mediated regulation of VDAC1 expression levels is associated with cell death induction. Biochim Biophys Acta (2014) 1843:2270–81. 10.1016/j.bbamcr.2014.03.021 24704533

[142] Huang L Han J Ben-Hail D He L Li B Chen Z A new fungal diterpene induces VDAC1-dependent apoptosis in Bax/Bak-deficient cells. J Biol Chem (2015) 290:23563–78. 10.1074/jbc.M115.648774 26253170 PMC4583032

[143] Ben-Hail D Begas-Shvartz R Shalev M Shteinfer-Kuzmine A Gruzman A Reina S Novel compounds targeting the mitochondrial protein VDAC1 inhibit apoptosis and protect against mitochondria dysfunction. J Biol Chem (2016) 291:24986–5003. 10.1074/jbc.M116.744284 27738100 PMC5122769

[144] Abu-Hamad S Arbel N Calo D Arzoine L Israelson A Keinan N The VDAC1 N-terminus is essential both for apoptosis and the protective effect of anti-apoptotic proteins. J Cell Sci (2009) 122:1906–16. 10.1242/jcs.040188 19461077

[145] Arzoine L Zilberberg N Ben-Romano R Shoshan-Barmatz V. Voltage-dependent anion channel 1-based peptides interact with hexokinase to prevent its anti-apoptotic activity. J Biol Chem (2009) 284:3946–55. 10.1074/jbc.M803614200 19049977

[146] Azoulay-Zohar H Israelson A Abu-Hamad S Shoshan-Barmatz V. In self-defence: hexokinase promotes voltage-dependent anion channel closure and prevents mitochondria-mediated apoptotic cell death. Biochem J (2004) 377:347–55. 10.1042/bj20031465 14561215 PMC1223882

[147] Mathupala SP Ko YH Pedersen PL Hexokinase II: cancer’s double-edged sword acting as both facilitator and gatekeeper of malignancy when bound to mitochondria. Oncogene (2006) 25:4777–86. 10.1038/sj.onc.1209603 16892090 PMC3385868

[148] Pastorino JG Hoek JB Shulga N. Activation of glycogen synthase kinase 3beta disrupts the binding of hexokinase II to mitochondria by phosphorylating voltage-dependent anion channel and potentiates chemotherapy-induced cytotoxicity. Cancer Res (2005) 65:10545–54. 10.1158/0008-5472.CAN-05-1925 16288047

[149] Pedersen PL Mathupala S Rempel A Geschwind JF Ko YH. Mitochondrial bound type II hexokinase: a key player in the growth and survival of many cancers and an ideal prospect for therapeutic intervention. Biochim Biophys Acta (2002) 1555:14–20. 10.1016/S0005-2728(02)00248-7 12206885

[150] Pastorino JG Shulga N Hoek JB. Mitochondrial binding of hexokinase II inhibits Bax-induced cytochrome c release and apoptosis. J Biol Chem (2002) 277:7610–8. 10.1074/jbc.M109950200 11751859

[151] Zaid H Abu-Hamad S Israelson A Nathan I Shoshan-Barmatz V. The voltage-dependent anion channel-1 modulates apoptotic cell death. Cell Death Differ (2005) 12:751–60. 10.1038/sj.cdd.4401599 15818409

[152] Abu-Hamad S Zaid H Israelson A Nahon E Shoshan-Barmatz V. Hexokinase-I protection against apoptotic cell death is mediated via interaction with the voltage-dependent anion channel-1: mapping the site of binding. J Biol Chem (2008) 283:13482–90. 10.1074/jbc.M708216200 18308720

[153] Shimizu S Ide T Yanagida T Tsujimoto Y Electrophysiological study of a novel large pore formed by Bax and the voltage-dependent anion channel that is permeable to cytochrome c. J Biol Chem (2000) 275:12321–5. 10766872 10.1074/jbc.275.16.12321

[154] Shimizu S Narita M Tsujimoto Y. Bcl-2 family proteins regulate the release of apoptogenic cytochrome c by the mitochondrial channel VDAC. Nature (1999) 399:483–7. 10.1038/20959 10365962

[155] Shi Y Chen J Weng C Chen R Zheng Y Chen Q Identification of the protein-protein contact site and interaction mode of human VDAC1 with Bcl-2 family proteins. Biochem Biophys Res Commun (2003) 305:989–96. 10.1016/S0006-291X(03)00871-4 12767928

[156] Westphal D Dewson G Czabotar PE Kluck RM. Molecular biology of Bax and Bak activation and action. Biochim Biophys Acta (2011) 1813:521–31. 10.1016/j.bbamcr.2010.12.019 21195116

[157] Anis Y Involvement of Ca2+ in the apoptotic process – friends or foes. Pathways (2006) 2:2–7.

[158] Gerasimenko JV Gerasimenko OV Palejwala A Tepikin AV Petersen OH Watson AJM Menadione-induced apoptosis: roles of cytosolic Ca2+ elevations and the mitochondrial permeability transition pore. J Cell Sci (2002) 115:485–97. 11861756 10.1242/jcs.115.3.485

[159] Boehning D Patterson RL Sedaghat L Glebova NO Kurosaki T Snyder SH. Cytochrome c binds to inositol (1,4,5) trisphosphate receptors, amplifying calcium-dependent apoptosis. Nat Cell Biol (2003) 5:1051–61. 10.1038/ncb1063 14608362

[160] Miyamoto S Howes AL Adams JW Dorn GW Brown JH. Ca2+ dysregulation induces mitochondrial depolarization and apoptosis: role of Na+/Ca2+ exchanger and AKT. J Biol Chem (2005) 280:38505–12. 10.1074/jbc.M505223200 16061478

[161] Rong Y Distelhorst CW. Bcl-2 protein family members: versatile regulators of calcium signaling in cell survival and apoptosis. Annu Rev Physiol (2008) 70:73–91. 10.1146/annurev.physiol.70.021507.105852 17680735

[162] Giorgi C Bonora M Sorrentino G Missiroli S Poletti F Suski JM p53 at the endoplasmic reticulum regulates apoptosis in a Ca2+-dependent manner. Proc Natl Acad Sci U S A (2015) 112:1779–84. 10.1073/pnas.1410723112 25624484 PMC4330769

[163] Borahay MA Kilic GS Yallampalli C Snyder RR Hankins GDV Al-Hendy A Simvastatin potently induces calcium-dependent apoptosis of human leiomyoma cells. J Biol Chem (2014) 289:35075–86. 10.1074/jbc.M114.583575 25359773 PMC4271198

[164] Hedgepeth SC Garcia MI Wagner LE Rodriguez AM Chintapalli SV Snyder RR The BRCA1 tumor suppressor binds to inositol 1,4,5-trisphosphate receptors to stimulate apoptotic calcium release. J Biol Chem (2015) 290:7304–13. 10.1074/jbc.M114.611186 25645916 PMC4358148

[165] Jiang N Kham SK Koh GS Suang Lim JY Ariffin H Chew FT Identification of prognostic protein biomarkers in childhood acute lymphoblastic leukemia (ALL). J Proteomics (2011) 74:843–57. 10.1016/j.jprot.2011.02.034 21396490

[166] Castagna A Antonioli P Astner H Hamdan M Righetti SC Perego P A proteomic approach to cisplatin resistance in the cervix squamous cell carcinoma cell line A431. Proteomics (2004) 4:3246–67. 10.1002/pmic.200400835 15378690

[167] Sharaf el dein O Gallerne C Brenner C Lemaire C. Increased expression of VDAC1 sensitizes carcinoma cells to apoptosis induced by DNA cross-linking agents. Biochem Pharmacol (2012) 83:1172–82. 10.1016/j.bcp.2012.01.017 22285227

[168] Jung JY Han CR Jeong YJ Kim HJ Lim HS Lee KH Epigallocatechin gallate inhibits nitric oxide-induced apoptosis in rat PC12 cells. Neurosci Lett (2007) 411:222–7. 10.1016/j.neulet.2006.09.089 17116366

[169] Voehringer DW Hirschberg DL Xiao J Lu Q Roederer M Lock CB Gene microarray identification of redox and mitochondrial elements that control resistance or sensitivity to apoptosis. Proc Natl Acad Sci U S A (2000) 97:2680–5. 10.1073/pnas.97.6.2680 10716996 PMC15989

[170] Cheng SL Liu RH Sheu JN Chen ST Sinchaikul S Tsay GJ. Toxicogenomics of A375 human malignant melanoma cells treated with arbutin. J Biomed Sci (2007) 14:87–105. 10.1007/s11373-006-9130-6 17103032

[171] Nawarak J Huang-Liu R Kao SH Liao HH Sinchaikul S Chen ST Proteomics analysis of A375 human malignant melanoma cells in response to arbutin treatment. Biochim Biophys Acta (2009) 1794:159–67. 10.1016/j.bbapap.2008.09.023 18996230

[172] Moin SM Panteva M Jameel S The hepatitis E virus Orf3 protein protects cells from mitochondrial depolarization and death. J Biol Chem (2007) 282:21124–33. 10.1074/jbc.M701696200 17488721 PMC2440810

[173] Liu Z Bengtsson S Krogh M Marquez M Nilsson S James P Somatostatin effects on the proteome of the LNCaP cell-line. Int J Oncol (2007) 30:1173–9. 17390019

[174] Tomasello F Messina A Lartigue L Schembri L Medina C Reina S Outer membrane VDAC1 controls permeability transition of the inner mitochondrial membrane in cellulo during stress-induced apoptosis. Cell Res (2009) 19:1363–76. 10.1038/cr.2009.98 19668262

[175] Liu S Ishikawa H Tsuyama N Li FJ Abroun S Otsuyama KI Increased susceptibility to apoptosis in CD45(+) myeloma cells accompanied by the increased expression of VDAC1. Oncogene (2006) 25:419–29. 10.1038/sj.onc.1208982 16247487

[176] Abu-Hamad S Sivan S Shoshan-Barmatz V. The expression level of the voltage-dependent anion channel controls life and death of the cell. Proc Natl Acad Sci U S A (2006) 103:5787–92. 10.1073/pnas.0600103103 16585511 PMC1458651

[177] Ghosh T Pandey N Maitra A Brahmachari SK Pillai B. A role for voltage-dependent anion channel VDAC1 in polyglutamine-mediated neuronal cell death. PLoS One (2007) 2:e1170. 10.1371/journal.pone.0001170 18000542 PMC2064964

[178] Godbole A Varghese J Sarin A Mathew MK. VDAC is a conserved element of death pathways in plant and animal systems. Biochim Biophys Acta (2003) 1642:87–96. 10.1016/S0167-4889(03)00102-2 12972297

[179] Shoshan-Barmatz V Keinan N Abu-Hamad S Tyomkin D Aram L. Apoptosis is regulated by the VDAC1 N-terminal region and by VDAC oligomerization: release of cytochrome c, AIF and Smac/Diablo. Biochim Biophys Acta (2010) 1797:1281–91. 10.1016/j.bbabio.2010.03.003 20214874

[180] Mellstrom B Savignac M Gomez-Villafuertes R Naranjo JR. Ca2+-operated transcriptional networks: molecular mechanisms and in vivo models. Physiol Rev (2008) 88:421–49. 10.1152/physrev.00041.2005 18391169

[181] Naranjo JR Mellström B Ca2+-dependent transcriptional control of Ca2+ homeostasis. J Biol Chem (2012) 287:31674–80. 10.1074/jbc.R112.384982 22822058 PMC3442502

[182] Arif T Vasilkovsky L Refaely Y Konson A Shoshan-Barmatz V. Silencing VDAC1 expression by siRNA inhibits cancer cell proliferation and tumor growth in vivo. Mol Ther Nucleic Acids (2014) 3:e159. 10.1038/mtna.2014.9 24781191 PMC4011124

[183] Shoshan-Barmatz V Ben-Hail D Admoni L Krelin Y Tripathi SS. The mitochondrial voltage-dependent anion channel 1 in tumor cells. Biochim Biophys Acta (2015) 1848:2547–75. 10.1016/j.bbamem.2014.10.040 25448878

[184] Koren I Raviv Z Shoshan-Barmatz V. Downregulation of voltage-dependent anion channel-1 expression by RNA interference prevents cancer cell growth in vivo. Cancer Biol Ther (2010) 9:1046–52. 10.4161/cbt.9.12.11879 20404552

[185] Arif T Kerlin Y Nakdimon I Benharroch D Paul A Dadon-Klein D VDAC1 is a molecular target in glioblastoma, with its depletion leading to reprogrammed metabolism and reversed oncogenic properties. Neuro Oncol (2017). 10.1093/neuonc/now297 28339833 PMC5570220

[186] Marchi S Pinton P. Alterations of calcium homeostasis in cancer cells. Curr Opin Pharmacol (2016) 29:1–6. 10.1016/j.coph.2016.03.002 27043073

[187] Cárdenas C Müller M McNeal A Lovy A Jaňa F Bustos G Selective vulnerability of cancer cells by inhibition of Ca2+ transfer from endoplasmic reticulum to mitochondria. Cell Rep (2016) 14:2313–24. 10.1016/j.celrep.2016.02.030 26947070 PMC4794382

[188] Rimessi A Patergnani S Bonora M Wieckowski MR Pinton P. Mitochondrial Ca(2+) remodeling is a prime factor in oncogenic behavior. Front Oncol (2015) 5:143. 10.3389/fonc.2015.00143 26161362 PMC4479728

[189] Sasaki K Donthamsetty R Heldak M Cho YE Scott BT Makino A. VDAC: old protein with new roles in diabetes. Am J Physiol Cell Physiol (2012) 303:C1055–60. 10.1152/ajpcell.00087.2012 22972802 PMC3492836

[190] Truong AH Murugesan S Youssef KD Makino A Mitochondrial ion channels in metabolic disease. In: Levitan I Dopico MA, editors. Vascular Ion Channels in Physiology and Disease. Switzerland: Springer International Publishing (2016). p. 397–419.

[191] Wang DM Li SQ Zhu XY Wang Y Wu WL Zhang XJ Protective effects of hesperidin against amyloid-beta (Abeta) induced neurotoxicity through the voltage dependent anion channel 1 (VDAC1)-mediated mitochondrial apoptotic pathway in PC12 cells. Neurochem Res (2013) 38:1034–44. 10.1007/s11064-013-1013-4 23475456

[192] Gonzalez S Berthelot J Jiner J Perrin-Tricaud C Fernando R Chrast R Blocking mitochondrial calcium release in Schwann cells prevents demyelinating neuropathies. J Clin Invest (2016) 126:2773 10.1172/JCI84505 PMC4922688 27111236

